# Long‐Term Effectiveness of Inpatient and Day‐Hospital Treatment of Eating Disorders in Departments of Psychosomatic Medicine and Psychotherapy in Germany

**DOI:** 10.1002/erv.70114

**Published:** 2026-04-18

**Authors:** Magdalena Pape, Stephan Herpertz, Stephan Doering, Henrik Kessler, Martina de Zwaan, Almut Zeeck, Armin Hartmann, Tobias Hofmann, Matthias Rose, Katrin Imbierowicz, Franziska Geiser, Ilona Croy, Kerstin Weidner, Jörg Rademacher, Silke Michalek, Eva Morawa, Yesim Erim, Eva‐Maria Skoda, Martin Teufel, Stanislav Heinzmann, Claas Lahmann, Eva Milena Johanne Peters, Johannes Kruse, Dirk von Boetticher, Christoph Herrmann‐Lingen, Mariel Nöhre, Ulrike Dinger, Hans‐Christoph Friederich, Alexander Niecke, Christian Albus, Rüdiger Zwerenz, Manfred Beutel, Casper Roenneberg, Peter Henningsen, Barbara Stein, Christiane Waller, Karsten Hake, Carsten Spitzer, Andreas Stengel, Stephan Zipfel, Katja Weimer, Harald Gündel, Aram Kehyayan

**Affiliations:** ^1^ Department of Psychosomatic Medicine and Psychotherapy LWL University Hospital Ruhr‐University Bochum Bochum Germany; ^2^ Department of Clinical Psychology and Psychotherapy University of Bamberg Bamberg Germany; ^3^ Department of Psychoanalysis and Psychotherapy Medical University of Vienna Vienna Austria; ^4^ Department of Psychosomatic Medicine and Psychotherapy Campus Fulda University of Marburg Marburg Germany; ^5^ Department of Psychosomatic Medicine and Psychotherapy Hannover Medical School Hannover Germany; ^6^ Department of Psychosomatic Medicine und Psychotherapy Medical Center—University of Freiburg Faculty of Medicine University of Freiburg Freiburg Germany; ^7^ Charité Center for Internal Medicine and Dermatology Department of Psychosomatic Medicine Charité‐Universitätsmedizin Berlin Corporate Member of Freie Universität Berlin and Humboldt‐Universität zu Berlin Berlin Germany; ^8^ Department of Psychosomatic Medicine and Psychotherapy DRK Kliniken Berlin Wiegmann Klinik Berlin Germany; ^9^ German Center for Mental Health (DZPG) Partner Site Berlin/Potsdam Berlin Germany; ^10^ Charité—Universitätsmedizin Berlin Center for Patient‐Centered Outcomes Research (CPCOR) Berlin Germany; ^11^ Department of Psychosomatic Medicine and Psychotherapy University Hospital Bonn University of Bonn Bonn Germany; ^12^ Department of Psychotherapy and Psychosomatic Medicine Faculty of Medicine and University Hospital Carl Gustav Carus TUD Dresden University of Technology Dresden Dresden Germany; ^13^ Department of Clinical Psychology Friedrich‐Schiller University Jena Germany; ^14^ Department of Psychosomatic Medicine and Psychotherapy LVR‐University Hospital Heinrich Heine University Düsseldorf Düsseldorf Germany; ^15^ Department of Psychosomatic Medicine and Psychotherapy University Hospital of Erlangen Friedrich‐Alexander University Erlangen‐Nuremberg Erlangen Germany; ^16^ Clinic of Psychosomatic Medicine and Psychotherapy LVR‐University Hospital University of Duisburg‐Essen Essen Germany; ^17^ Germany and Center for Translational Neuro‐ and Behavioral Sciences University of Duisburg‐Essen Essen Germany; ^18^ Department of Psychosomatic Medicine and Psychotherapy Justus‐Liebig University of Giessen Germany and Department of Psychosomatic Medicine and Psychotherapy Philipps‐University of Marburg Marburg Germany; ^19^ Department of Psychosomatic Medicine and Psychotherapy University Medical Centre Göttingen Göttingen Germany; ^20^ Department of General Internal Medicine and Psychosomatics University Hospital Heidelberg University Heidelberg Germany; ^21^ Department of Psychosomatic Medicine and Psychotherapy University of Cologne Faculty of Medicine and University Hospital Cologne Köln Germany; ^22^ Department of Psychosomatic Medicine and Psychotherapy University Medical Center of the Johannes Gutenberg University Mainz Mainz Germany; ^23^ Department of Psychosomatic Medicine and Psychotherapy University Hospital Technical University of Munich Munich Germany; ^24^ Department of Psychosomatic Medicine and Psychotherapy Paracelsus Medical University Nuremberg General Hospital Nuremberg Germany; ^25^ Department of Psychosomatic Medicine and Psychotherapy University Medical Center Rostock Rostock Germany; ^26^ Internal Medicine VI, Psychosomatic Medicine and Psychotherapy University Hospital Tübingen Tübingen Germany; ^27^ German Center for Mental Health, Site Tübingen Tübingen Germany; ^28^ Clinic for Psychosomatic Medicine and Psychotherapy Klinikum Stuttgart Stuttgart Germany; ^29^ Department of Psychosomatic Medicine and Psychotherapy Ulm University Medical Center Ulm Germany

**Keywords:** eating disorders, effectiveness, Germany, psychosomatic, psychotherapy

## Abstract

**Background:**

There is ample evidence on the efficacy of different therapeutic approaches in treating eating disorders. Yet, studies on the effectiveness of inpatient/day‐hospital treatment of eating disorders are rare.

**Method:**

Data were retrieved from a naturalistic multi‐center effectiveness study of inpatient and day‐hospital treatment in departments of psychosomatic medicine and psychotherapy in Germany (MEPP). *N* = 151 patients with Anorexia Nervosa (AN) and atypical AN, as well as *n* = 120 patients with Bulimia Nervosa (BN) and atypical BN were included. Eating disorder pathology (EDE‐Q) and BMI were assessed on admission, at discharge, and 1 year after discharge. Changes over time were analysed in both outcomes. Treatment success was assessed individually using reliable change (RC) indices. Regression analyses were used to identify predictors of RC.

**Results:**

EDE‐Q scores decreased in both patient groups. One year after discharge, RC was estimated in 33.8% of patients with AN and 43.3% of patients with BN. 24.2% of patients with AN and 54.3% with BN showed partial remission.

**Discussion:**

Results from this multi‐center effectiveness study are comparable to those of former national and European studies. Based on the results of individual treatment success, recommendations for inpatient/day‐hospital treatment can be derived.

**Trial Registration:**

The study was registered at the German Clinical Trials Register (www.drks.de; ID: DRKS00016412)

## Highlights

- Effectiveness of inpatient and day‐hospital treatment was assessed in 19 German university hospital departments of psychosomatic medicine and psychotherapy in a large multi‐center study.
- This article reports treatment effectiveness for a subgroup of patients suffering from eating disorders Anorexia Nerosa and Bulimia Nervosa.
- Treatment was found to be effective, with stable effects over the 1‐year follow‐up period.

## 1 Introduction

Eating disorders (EDs), that is Anorexia Nervosa (AN) and Bulimia Nervosa (BN), are severe mental disorders that cause high direct and indirect costs for health care systems and society (Krauth et al. 2002). AN is characterised by a self‐induced and sustained weight loss, subsequently resulting in underweight (World Health Organization, WHO 2022). It is associated with a dread of weight gain and a distortion of body image accompanied by an over‐evaluating of thinness. AN typically develops in the peripubertal period of adolescents (Treasure et al. 2015). In Germany, the 12‐month prevalence for AN is about 0.7% of the adult population, with higher prevalence rates in women (1.1%) compared to men (0.3%) (Jacobi et al. 2014). Due to the somatic consequences of extreme underweight and suicidality of affected individuals, AN has the highest (5.35) standardized mortality rate of mental disorders (Fichter et al. 2016). About 25% of the deaths are from suicide (Attia and Walsh 2025). Some individuals are affected by atypical AN, they meet most diagnostic criteria of AN, yet are usually still within the normal weight range (BMI ≥ 18.5 kg/m^2^) (Moskowitz and Weiselberg 2017).

Bulimia Nervosa (BN) is characterised by recurrent (at least one per week) binge eating episodes over a period of 3 months (American Psychiatric Association 2018). During binge eating episodes, affected people experience a lack of control over their eating behaviour, subsequently consuming large amounts of food in short periods of time. In addition, people with BN try to compensate for these episodes and avoid weight gain by compensatory behaviour, for example self‐induced vomiting or excessive exercise. Affected people usually experience a high level of distress concerning their weight and shape. The 12‐month prevalence of BN in Germany is 0.2%, with higher rates for women (0.3%) compared to men (0.1%), too (Jacobi et al. 2014). As for AN, especially the group of younger adults are at risk. Individuals affected by atypical BN fulfill some of the features of BN, but not all required diagnostic criteria.

EDs are highly comorbid with other mental disorders, especially depression, anxiety disorders, and personality disorders, which aggravate recovery (Juli et al. 2023). The frequency of binge eating and mental comorbidity are negative predictors for long‐term recovery of EDs (Franko et al. 2018). There is a large amount of evidence for the efficacy of behavioural and cognitive‐behavioural therapy in successfully treating BN and AN (Russell et al. 2023; T. D. Wade 2019). Also, other treatment approaches, like family‐based treatment, focal psychodynamic therapy, specialist supportive clinical management (SSCM) and the Maudsley model of anorexia nervosa treatment of adults (MANTRA) have proven their efficacy (Attia and Walsh 2025; Zeeck et al. 2018; Zipfel et al. 2015). However, efficacy studies are designed and conducted under standardized conditions that hardly reflect the reality of routine health care.

To date, only a few studies have analysed the effectiveness of inpatient and day‐hospital ED treatment in a naturalistic setting. In Germany, inpatient treatment is recommended, if the severity and/or chronicity of ED pathology is high, or the patients exhibit a high degree of comorbidity with other mental disorders (Brockmeyer 2018). Given the physical risk of sustained underweight, inpatient treatment is more often necessary for patients with AN, that is, adult patients. Day‐hospital treatment is recommended as a “step‐down” approach following inpatient treatment and focuses on the integration of restructured eating behaviours into daily life. Tagay et al. (2011) consecutively recruited *N* = 128 patients with either AN or BN diagnoses at a university hospital in Germany. After 78 days (on average) of inpatient treatment, patients reported a significant reduction of ED symptoms and general psychopathology, as well as improvements in quality of life and personal resources. Schlegl et al. (2014) reported results from inpatient treatment of *N* = 436 patients with AN in a hospital in southern Germany. An advantage of that publication is that they examined effectiveness on an intra‐individual level, revealing that 35.6% of the patients showed a clinically significant improvement in ED psychopathology on discharge. Results from the same research group revealed that 43.2% of *N* = 295 consecutively admitted patients with BN showed a clinically significant improvement in ED psychopathology at discharge (Diedrich et al. 2018). However, in order to improve representativeness and reduce potential biases (such as the environment of the hospital), multi‐center studies are necessary. About 25 years ago, (Kächele 1999) conducted a large multi‐center study analysing the effectiveness of psychodynamic therapy in Germany. The results indicated an improvement in ED psychopathology. 2.5 years after admission, the remission rates were about 25% for BN and 33% for AN (Kächele et al. 2001). To date, inpatient/day‐hospital treatment in psychosomatic and psychotherapy departments in Germany combines different psychotherapeutic approaches (e.g., focal psychodynamic therapy, CBT, supervised meals) within a multimodal therapy concept. Therefore, there is a need for an update on the effectiveness of ED treatment in departments of psychosomatic medicine and psychotherapy.

Taken together, EDs are severe mental disorders. There exists ample evidence on the efficacy of ED treatment from different therapeutic approaches, although treatment results still remain unsatisfactory. Yet, evidence from naturalistic studies concerning the effectiveness of current inpatient/day‐hospital treatment in Germany is rare. Within a multi‐center study of 19 German university departments of psychosomatic medicine and psychotherapy, *n* = 271 patients with (atypical) AN (ICD‐10: F50.0 and F50.1) or (atypical)BN (ICD‐10: F50.2 and F50.3) were consecutively included and examined by combining group and intra‐individual level statistical analyses.

## 2 Materials and Methods

Data on the subgroup of patients with EDs were retrieved from the above‐mentioned multi‐center study assessing the effectiveness of psychosomatic treatment in Germany (MEPP study). The overall study sample comprised *N* = 2094 adult patients, a detailed description of the sample and overall effectiveness results has been published elsewhere (Doering, Herpertz, Pape, et al. 2023; Doering, Herpertz, Hofmann, et al. 2023; Kessler et al. 2025). The study was approved by the Ethics Committee of the Ruhr‐University Bochum on October 17, 2018 (ID: 18‐6388) and registered at the German Clinical Trials Register (DRKS‐ID: DRKS00016412). The study was conducted across 19 German university departments of Psychosomatic Medicine and Psychotherapy.

### 2.1 Participants

**FIGURE 1:**
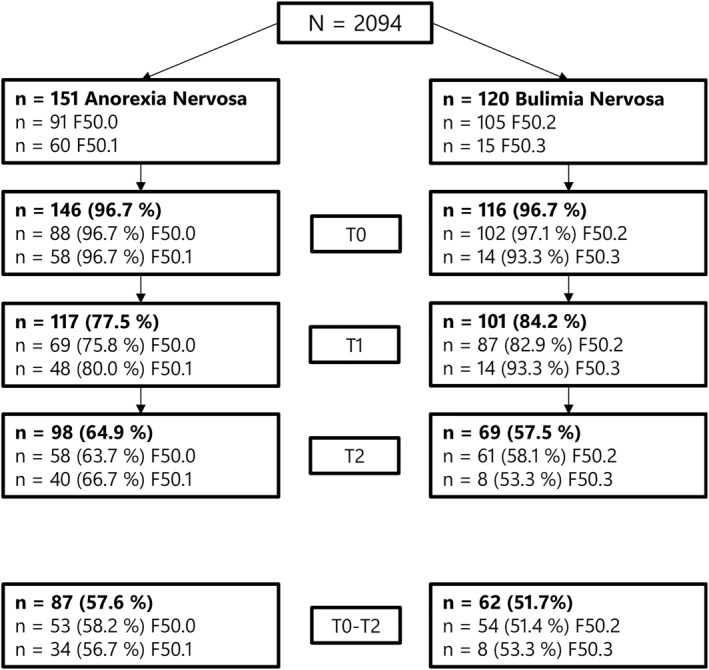
Flowchart of the study population separated for anorexia nervosa and bulimia nervosa with regard to present primary outcome data at each assessment point.

Between January 2019 and December 2020, a total of *N* = 2094 patients were included consecutively, of whom *n* = 151 were diagnosed with (atypical) AN (F50.0 + F50.1) and *n* = 120 were diagnosed with (atypical) BN (F50.2 + F50.3). All patients provided written informed consent and were screened for eligibility by trained clinicians. Inclusion criteria were age ≥ 18 years, sufficient knowledge of the German language, and regular (non‐emergency) admission for inpatient or day‐hospital treatment. Exclusion criteria were clinically relevant organic brain disorders, acute psychotic disorders, and current substance use disorders (excluding nicotine). Data were assessed at three measurement points: on admission (T0), during the last week before discharge (T1), and 1 year after discharge (follow‐up, T2). T0 and T1 assessments were conducted on site. The T2 assessment was either conducted online or relevant questionnaires were sent by post. Figure 1 displays a flowchart of the study population of ED patients with regard to primary outcome data EDE‐Q (Hilbert and Tuschen‐Caffier 2016) at each assessment point. Within the group of patients with AN, *n* = 124 (82.1%) underwent inpatient treatment. Fewer patients with BN (*n* = 92, 76.7%) underwent inpatient treatment. *N* = 132 (87.4%) of patients with AN and *n* = 107 (89.2%) of patients with BN were discharged regularly.

### 2.2 Treatment

Due to the naturalistic setting, patients received optimised treatment as usual at the participating German university hospital departments of Psychosomatic Medicine and Psychotherapy. The multimodal treatment comprised psychodynamic, CBT and systemic psychotherapeutic elements, as well as other therapy approaches, such as movement therapy, with a high dose of 15–20 h per week of individual and group treatments (Doering, Herpertz, Hofmann, et al. 2023). The treatment at the participating university hospitals was based on the S3 guidelines of the German Association of the Scientific Medical Societies (AWMF) (Brockmeyer 2018). For inpatient ED treatment in Germany, the consensus guideline recommends flexible and individualised disorder‐oriented treatment. For patients with AN, the guidelines recommend significant weight gain, monitored by contracts between patients and therapists regarding a target weight and a minimum of weight gain per week. To ensure sufficient calorie intake, food intake should be monitored and if necessary adjusted by liquid foods. In addition, it is recommended to focus on ambivalences concerning weight gain and enhance motivation to change, as well as addressing body image distortion. In cases of severe chronicity, treatment should focus on an enhancement and stabilisation of quality of life.

For patients with BN, the guidelines recommend a reduction of binge eating episodes and compensatory behaviours. Eating behaviour should be restructured and monitored, dysfunctional beliefs about the importance of body and weight should be addressed. For all EDs, underlying psychological aspects and comorbid symptoms should be considered. Due to the naturalistic setting, it is not possible to control for discrepancies from the recommendations.

### 2.3 Instruments

At T0 and T1, the patients' weight and height were measured. In addition, patients completed self‐report measures at each of the three assessments (T0–T2). On admission, standardized structured interviews were conducted to provide valid diagnoses. The interviews were conducted by clinicians (psychologists, or medical professionals) with expertise in diagnosing mental disorders. The interviewers were trained to carry out the following structured interviews.

#### 2.3.1 Mental Disorders

The short version of the Diagnostic Interview for Mental Disorders (“Diagnostisches Interview bei psychischen Störungen—Mini‐DIPS”) was used to assess EDs and comorbid mental disorders based on the *Diagnostic and Statistical Manual of Mental Disorders* (DSM‐5) criteria, which can be translated into ICD‐10 diagnoses (Margraf et al. 2017; Margraf and Cwik 2020). The Mini‐DIPS showed good psychometric properties with high comparability to the long version of the interview and very good interrater reliability (Yules Y‐coefficient = 1.0).

#### 2.3.2 Personality Disorders

Personality disorders were assessed using the German version of the Structured Interview for DSM‐IV axis II (Fydrich et al. 1997). The interrater reliability of 22 raters was reported with Fleiss *κ* = 0.847.

#### 2.3.3 Body Mass Index (BMI)

At T0 and T1, weight was assessed at the hospital and BMI (kg/m^2^) was calculated. At the T2 follow‐up assessment, patients self‐reported their current body weight either online or via paper‐pencil questionnaires.

#### 2.3.4 Eating Pathology

The German version of the Eating Disorder Examination Questionnaire (EDE‐Q) was used to assess eating pathology (Hilbert and Tuschen‐Caffier 2016). The self‐report questionnaire assesses symptoms over the past 28 days and consists of 22 items, which are scored on a 7‐point forced‐choice rating scheme ranging from 0 (“never or non‐existent”) to 6 (“daily or extremely pronounced”). A global mean score can be calculated, as well as four subscale mean scores referring to: restraint eating (5 items), eating concern (5 items), weight concern (5 items) and shape concern (8 items). Six additional items assess frequencies of specific eating disorder behaviours, including the frequency of binge eating and compensatory behaviour within the last 28 days, but do not contribute to the score. The test‐statistic evaluation of the EDE‐Q showed good psychometric properties (Hilbert et al. 2007). In the present sample, internal consistency at admission (T0) was excellent for the global score (*α* = 0.959) and the subscale shape concern (*α* = 0.925), and good for the subscales restraint (*α* = 0.885), eating concern (*α* = 0.873) and weight concern (*α* = 0.856).

#### 2.3.5 AN and BN Severity and Remission

According to the DSM‐5, four AN severity levels can be defined: *mild* (BMI > 17.0 kg/m^2^), *moderate* (BMI range: 16–16.99 kg/m^2^), *severe* (BMI rang: 15–15.99 kg/m^2^) and *extreme* (BMI < 15.0 kg/m^2^) (American Psychiatric Association 2018). While BN severity degrees are defined by the frequency of compensatory behaviour per week: *mild:* 1–3, *moderate*: 4–7, *severe*: 8–13 and *extreme:* > 13 (American Psychiatric Association 2018). In this manuscript partial remission was defined by a normalisation of body weight for patients with AN (BMI ≥ 18.5 kg/m^2^) and a reduction of binge eating and compensatory behaviour below the criterion level (< 1/week).

### 2.4 Statistical Analysis

All analyses were conducted with IBM SPSS statistics for Windows (Version 29.0, Armonk, NY: IBM Corp.) and Microsoft Excel (Version 16.0, Microsoft Corporation). The groups of atypical AN and AN, as well as atypical BN and BN were collapsed for analyses. Descriptive analyses were conducted using percentages and frequencies for categorical variables, as well as means, standard deviations and median for continuous variables. The Last Observation Carried Forward (LOCF) method was used to replace missing values of the outcome measures. To examine changes in eating disorder psychopathology (EDE‐Q mean score) in patients with AN and BN, respectively, *univariate repeated measures analyses of variances* (rANOVAs) were conducted. While changes in BMI, binge eating episodes and compensatory behaviour as indicators for severity and remission were analysed by excluding atypical cases. Effect sizes (*ηp* ^2^) and confidence intervals were conducted. To correct for violations of sphericity, the Greenhouse–Geisser adjustment was used. Bonferroni‐adjusted post hoc tests were conducted to compare results between the three measurement points.

In addition, treatment success was examined on an individual basis by calculating the *reliable change index (RCI* (Jacobson and Truax 1991) for the EDE‐Q mean score:

$$SE_{M}=s\;\sqrt{1-r}$$

$$S_{\text{DIFF}}=\sqrt{2\left(SE_{m}^{2}\right)}$$

$$\text{RCI}=S_{\text{DIFF}}\times 1.96$$

$$\text{DIFF}=x_{0}\;–\;x_{2}$$

SE_*M*_ = standard error for measurement

*s* = standard deviation of reference group for EDE‐Q

*r* = Cronbach's alpha of the measurement

*S*_DIFF_ = standard errors of measurements of the difference scores

RCI = Reliable Change Index (absolute value of *DIFF* required for a reliable change).

DIFF = individual difference score between T0 and T2

In the following, differences in sociodemographic data, BMI, comorbidities, frequency of binge eating and compensatory behaviour, subscales of the EDE‐Q at baseline, as well as treatment duration (days) between patients *with reliable change (RC)* and *without RC (NRC)* were calculated by using *Chi‐square* distributions for categorical variables and Bonferroni‐adjusted independent *t*‐tests for metrically scaled variables. Effect sizes for *Chi‐square* (*ϕ*) and *t‐*tests (Cohens *d*) were conducted. Variables with significant group differences were subsequently included into stepwise binary logistic regression analyses to identify predictors of RC (vs. NRC). The backward likelihood ratio method was used to identify the strongest predictor of RC while checking for significant improvement of the regression model when eliminating predictors.

## 3 Results

Sociodemographic data and comorbidities of the sample are presented in Table 1.

**TABLE 1:** Sociodemographic data and comorbidities.

	Overall (*N* = 271)	Anorexia nervosa (*N* = 151)	Bulimia nervosa (*N* = 120)
Sociodemographic data			
Sex			
Female	*n* = 252 (93.0%)	*n* = 140 (92.7%)	*n* = 112 (93.3%)
Male	*n* = 17 (6.3%)	*n* = 11 (7.3%)	*n* = 6 (5.0%)
Age (years)	*M* = 30.36, SD = 10.84	*M* = 28.79, SD = 10.36	*M* = 32.37, SD = 11.15
Marital status			
Married/in a relationship	*n* = 103 (38.0%)	*n* = 53 (35.1%)	*n* = 50 (41.7%)
Education^a^	*n* = 150 (55.4%)	*n* = 82 (54.3%)	*n* = 68 (56.7%)
Employment^b^	*n* = 105 (38.7%)	*n* = 53 (35.1%)	*n* = 52 (43.3%)
Housekeeping	*n* = 4 (1.5%)	*n* = 2 (1.3%)	*n* = 2 (1.7%)
Comorbidities			
Depression	*n* = 220 (81.2%)	*n* = 119 (78.8%)	*n* = 101 (84.2%)
Anxiety disorders	*n* = 126 (46.5%)	*n* = 65 (43.0%)	*n* = 61 (50.8%)
PTSD	*n* = 51 (18.8%)	*n* = 22 (14.6%)	*n* = 29 (24.2%)
Somatoform disorders	*n* = 63 (23.2%)	*n* = 35 (23.2%)	*n* = 28 (23.3%)
Personality disorders	*n* = 152 (56.1%)	*n* = 74 (49.0%)	*n* = 78 (65.0%)

Abbreviation: PTSD, Post Traumatic Stress Disorder.

^a^ Education: number of participants with high school or university degree.

^b^ Employment: number of participants that are currently employed.

Treatment duration varied substantially, with a minimum of 2 days and a maximum of 238 days in patients with AN (*M* = 64.47, SD = 38.84, Md = 58) and a minimum of 5 days and a maximum of 97 days in patients with BN (*M* = 50.04, SD = 19.53, Md = 52).

**FIGURE 2:**
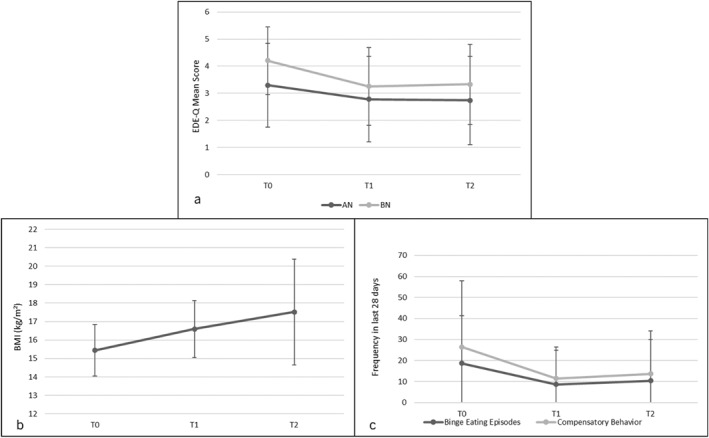
(a–c): (a) eating pathology as measured by the EDE‐Q sum score (M and SD) over time separated for AN and BN, (b) BMI over time in patients with AN (F50.0, *n* = 88), (c) number of binge eating episodes and compensatory behaviour over time in patients with BN (F50.2, *n* = 97). T0: admission; T1: discharge; T2: 1‐year follow‐up.

Figure 2a illustrates trends in eating pathology in patients with AN and BN over the three assessments (T0–T2). Descriptive data is presented in Table 2.

**TABLE 2:** Descriptive data about trends in eating pathology (T0–T2).

Anorexia nervosa			Bulimia nervosa	
	EDE‐Q		EDE‐Q	
	Mean	SD	Mean	SD
Admission (T0)	3.30	1.55	4.20	1.24
Discharge (T1)	2.78	1.58	3.25	1.44
1‐year follow‐up (T2)	2.73	1.63	3.33	1.47

	**BMI (kg/m** ^**2**^ **)**		**Binge eating episodes**		**Compensatory behaviour**	
	**Mean**	**SD**	**Mean**	**SD**	**Mean**	**SD**
Admission (T0)	15.43	1.39	18.75	22.64	26.43	31.46
Discharge (T1)	16.59	1.54	8.67	16.31	11.49	14.83
1‐year follow‐up (T2)	17.52	2.86	10.38	19.49	13.68	20.35

In patients with AN, a repeated measures ANOVA revealed a decrease of eating pathology over time, F(1.720, 249.47) = 24.52, p<0.001, *ηp* ^2^ = 0.145. Bonferroni‐adjusted post‐hoc tests revealed a significant difference (p<0.001) between T0 and T1 (*M*_Diff_ = −0.52, 95%–CI [−0.73, −0.30]), as well as between T0 and T2 (*M*_Diff_ = −0.56, 95%–CI [−0.81, −0.31]).

In patients with BN, a repeated measures ANOVA also revealed a decrease of eating pathology over time, F(2, 230) = 39.20, p<0.001, *ηp* ^2^ = 0.254. Bonferroni‐adjusted post‐hoc tests revealed a significant difference (p<0.001) between T0 and T1 (*M*_Diff_ = −0.95, 95%–CI [−1.23, −0.67]), as well as between T0 and T2 (*M*_Diff_ = −0.82, 95%–CI [−0.56, −1.18]).

Figure 2b illustrates changes in BMI in patients with AN (F50.0) over the three assessments (T0–T2). Results from a repeated measures ANOVA revealed a statistically significant increase in BMI over time, F(1.343, 116.883) = 36.64, p<.001, *ηp* ^2^ = 0.296. Bonferroni‐adjusted post‐hoc tests revealed a significant difference (p<.001) between T0 and T1 (*M*_Diff_ = 1.16, 95%–CI [0.83, 1.49]), as well as between T0 and T2 (*M*_Diff_ = 2.08, 95%‐CI [1.39, 2.77]). Moreover, BMI increased significantly (p=.005) between T1 and T2 (*M*_Diff_ = 0.92, 95%–CI [0.23, 1.62]. At the 1 year follow‐up assessment, the number of patients with *extreme underweight* (BMI < 15.0 kg/m^2^) decreased from *n* = 38 (41.8%) to *n* = 10 (11.0%), while the number of patients with *mild underweight* (BMI > 17.0 kg/m^2^) increased from *n* = 13 (14.3%) to *n* = 30 (33.0%). Moreover, at T2 *n* = 22 (24.2%) patients with AN had a BMI ≥ 18.5 kg/m^2^, indicating *normal weight*.

Figure 2c illustrates binge eating and compensatory behaviour in the last 28 days in patients with BN (F50.2) over the three assessments (T0–T2). Results from repeated measures ANOVAs revealed statistically significant decreases of compensatory behaviour (F(1.729, 169.394) = 19.90, p<0.001, *ηp* ^2^ = 0.169) and binge eating over time, F(1.719, 164.998) = 20.72, p<.001, *ηp* ^2^ = 0.177. Bonferroni‐adjusted post‐hoc tests revealed a significant difference in binge eating episodes (p<0.001) between T0 and T1 (*M*_Diff_ = −10.83, 95%–CI [−15.64, −6.01]), as well as between T0 and T2 (*M*_Diff_ = −9.27, 95%–CI [−14.17, −4.37]). In addition, a significant difference (p<0.001) between T0 and T1 (*M*_Diff_ = −14.83, 95%–CI [8.14, 21.52]), as well as between T0 and T2 in compensatory behaviours was detected (*M*_Diff_ = −12.73, 95%–CI [5.86, 19.60]). No significant differences were found between T1 and T2 for binge eating and compensatory behaviour respectively.

The number of patients with *severe* or *extreme* severity (frequency of compensatory behaviour/week > 7) decreased from *n* = 27 (25.7%) on admission to *n* = 12 (11.4%) at the follow‐up assessment. One year after discharge, *N* = 57 (54.3%) of the patients with BN reported less than 1 binge eating/week, subsequently not fulfilling the BN diagnosis.

Based on standard deviations of a German reference group (Hilbert and Tuschen‐Caffier 2016) and a Cronbach's alpha of 0.959, the RCI was calculated to be 0.803 for patients with AN and 0.881 for patients with BN. It describes the required absolute change in the value of the EDE‐Q mean scores to achieve a RC. Based on a difference score of 0.803 and 0.881, respectively, between T0 and T2, the percentage of patients with AN with a RC of the EDE‐Q mean score was *n* = 51 (33.8%), whereas the percentage of patients with BN with a RC of the EDE‐Q mean score was *n* = 52 (43.3%). Descriptive data on differences between patients with and without RC in both patient groups are presented in Tables 3 and 4.

**TABLE 3:** Sociodemographic data, comorbidities, BMI, treatment duration and EDE‐Q subscale scores data RC versus NRC in patients with Anorexia Nervosa (AN) on admission.

	AN with RC (*n* = 51)	AN with NRC (*N* = 95)	RC versus NRC^a^
Sociodemographic data			
Sex			
Female	*n* = 49 (96.1%)	*n* = 86 (90.5%)	*χ*(2) = 1.92, p=.383
Male	*n* = 2 (3.9%)	*n* = 9 (9.5%)	
Age (years)	*M* = 28.04, SD = 9.91	*M* = 28.69, SD = 10.08	*t*(144) = −0.38, p=4.95
Marital status			
Married/in a relationship	*n* = 15 (29.4%)	*n* = 37 (39.0%)	*χ*(2) = 1.84, p=0.398
Single	*n* = 36 (70.6%)	*n* = 58 (61.0%)	
Education^b^	*n* = 33 (64.7%)	*n* = 49 (51.6%)	*χ*(2) = 8.45, p=0.015, *ϕ* = 0.24
Employment^c^	*n* = 21 (41.2%)	*n* = 32 (33.7%)	*χ*(2) = 3.61, p=0.164
Comorbidities			
Depression	*n* = 37 (72.5%)	*n* = 78 (82.1%)	*χ*(2) = 1.82, p=0.403
Anxiety disorders	*n* = 22 (43.1%)	*n* = 39 (41.1%)	*χ*(2) = 2.94, p=0.230
PTSD	*n* = 6 (11.8%)	*n* = 14 (14.7%)	*χ*(2) = 2.92, p=0.232
Somatoform disorders	*n* = 5 (9.8%)	*n* = 29 (30.5%)	*χ*(2) = 8.03, p=0.018, *ϕ* = 0.23
Personality disorders	*n* = 28 (54.9%)	*n* = 42 (44.2%)	*χ*(2) = 3.51, p=0.173
BMI (kg/m^2^)	*M* = 16.84, SD = 2.66	*M* = 17.55, SD = 3.72	*t*(129.79) = −1.34, p=1.28
Treatment duration (days)	*M* = 72.70, SD = 35.7	*M* = 61.56, SD = 40.2	*t*(124) = 1.54, p=0.79
EDE‐Q subscales			
Restraint eating	*M* = 3.68, SD = 1.75	*M* = 2.62, SD = 2.08	*t*(116.45) = 3.19, p=0.014, *d* = 0.54
Eating concern	*M* = 3.53, SD = 1.41	*M* = 2.33, SD = 1.81	*t*(120.29) = 4.33, p<0.001, *d* = 0.72
Weight concern	*M* = 3.93, SD = 1.27	*M* = 2.93, SD = 1.76	*t*(126.54) = 3.86, p<0.001, *d* = 0.62
Shape concern	*M* = 4.46, SD = 1.09	*M* = 3.39, SD = 1.61	*t*(132.12) = 4.65, p<0.001, *d* = 0.74

Abbreviation: PTSD, Post Traumatic Stress Disorder.

^a^ Bonferroni‐adjusted *p*‐values.

^b^ Education: number of participants with high school or university degree.

^c^ Employment: number of participants that are currently employed (including housekeeping).

**TABLE 4:** Sociodemographic data, comorbidities, binge eating, compensatory behaviour, treatment duration and EDE‐Q subscale scores data RC versus NRC in patients with Bulimia Nervosa (BN) on admission.

	BN with RC (*n* = 52)	BN with NRC (*N* = 64)	RC versus NRC^a^
Sociodemographic data			
Sex			
Female	*n* = 48 (92.3%)	*n* = 62 (96.9%)	(2) = 1.35, p=0.509
Male	*n* = 4 (7.7%)	*n* = 2 (3.1%)	
Age (years)	*M* = 29.12, SD = 9.21	*M* = 34.41, SD = 11.47	*t*(113.99) = 2.76, p=0.056
Marital status			
Married/in a relationship	*n* = 22 (42.3%)	*n* = 27 (42.2%)	*χ*(2) = 0.47, p=0.789
Single	*n* = 30 (57.7%)	*n* = 37 (57.8%)	
Education^b^	*n* = 33 (63.4%)	*n* = 35 (54.7%)	*χ*(2) = 6.31, p=0.043, *ϕ* = 0.23
Employment^c^	*n* = 21 (40.4%)	*n* = 31 (48.4%)	*χ*(2) = 3.92, p=0.141
Comorbidities			
Depression	*n* = 42 (80.8%)	*n* = 56 (87.5%)	*χ*(2) = 1.24, p=0.539
Anxiety disorders	*n* = 21 (40.4%)	*n* = 39 (60.9%)	*χ*(2) = 5.95, p=0.049, *ϕ* = 0.22
PTSD	*n* = 7 (13.5%)	*n* = 20 (31.3%)	*χ*(2) = 6.46, p=0.040, *ϕ* = 0.23
Somatoform disorders	*n* = 10 (19.2%)	*n* = 18 (28.1%)	*χ*(2) = 2.53, p=0.283
Personality disorders	*n* = 34 (65.4%)	*n* = 43 (67.2%)	*χ*(2) = 2.95, p=0.229
Binge eating episodes (past 28 days)	*M* = 16.00. SD = 16.72	*M* = 21.08, SD = 26.58	*t(*109) = 1.18*,* p=1.92
Compensatory behaviour (past 28 days)	*M* = 23.23, SD = 23.04	*M* = 27.90, SD = 38.87	*t(*99.72) = 0.79*,* p=3.456
Treatment duration (days)	*M* = 52.28, SD = 16.05	*M* = 49.81, SD = 20.58	*t*(95) = 0.64, p=4.216
EDE‐Q subscales			
Restraint eating	*M* = 3.98, SD = 1.79	*M* = 3.45, SD = 1.79	*t*(112) = 1.58, p=0.936
Eating concern	*M* = 3.84, SD = 1.49	*M* = 3.50, SD = 1.62	*t*(111) = 1.17, p=1.96
Weight concern	*M* = 4.64, SD = 1.20	*M* = 3.89, SD = 1.53	*t*(112) = 2.89, p=0.032, *d* = 0.54
Shape concern	*M* = 4.98, SD = 1.04	*M* = 4.65, SD = 1.28	*t*(113) = 1.52, p=0.528

Abbreviation: PTSD, Post Traumatic Stress Disorder.

^a^ Bonferroni‐adjusted *p*‐values.

^b^ Education: number of participants with high school or university degree.

^c^ Employment: number of participants that are currently employed (including housekeeping).

In the AN sample, more patients with RC had a high school or university degree compared to patients with no RC, *χ*(2) = 8.45, p=0.015, *ϕ* = 0.24. Whereas less patients with RC were diagnosed with somatoform disorders compared to patients with NRC, *χ*(2) = 8.03, p=0.018, *ϕ* = 0.23. With regard to the EDE‐Q subscales, patients with RC reported higher *restraint eating* compared to patients with NRC, *t* (116.45) = 3.19, p=.014, *d* = 0.54; as well as higher *eating concern* compared to patients with NRC, *t* (120.29) = 4.33, p<0.001, *d* = 0.72. Patients with RC also reported higher *weight concern* compared to patients with NRC, *t* (126.54) = 3.86, p<0.001, *d* = 0.62; and higher *shape concern* compared to patients with NRC on admission, *t* (132.12) = 4.65, p<0.001, *d* = 0.74. No further differences on sociodemographic data, comorbidities, BMI and treatment duration were observed.

Variables with significant group differences were subsequently included as independent variables into a stepwise binary logistic regression analysis with RC versus NRC as dependent variable. The backward likelihood ratio method was used to identify significant predictors of therapy success. The final model was statistically significant, *χ* ^2^(2) = 21.88, p<0.001, resulting in an acceptable amount of explained variances (Nagelkerke's *R* ^2^ = 0.203). The overall percentage of accuracy in classification was 67.9%. Of the five independent variables, two were included in the final model and significantly predicted RC: the EDE‐Q subscale *shape concern* (p<.001) and somatoform disorder (p=.014). Higher *shape concern* on admission enhanced the likelihood for RC, OR = 1.66 (95%–CI [1.25, 2.20]), while being diagnosed with somatoform disorders reduced the likelihood for RC, OR = 0.26 (95%–CI [0.09, 0.77]). With an increase of 1.38 points in the *shape concern* subscale on admission, the odds for RC doubled, while patients who were diagnosed with somatoform disorders had a 3.80 higher probability for NRC.

In the BN sample, also more patients with RC had a high school or university degree than patients with NRC, *χ*(2) = 6.31*,* p=.043, *ϕ* = 0.23. Whereas fewer patients with RC were diagnosed with anxiety disorders compared to patients with NRC, *χ*(2) = 5.95, p=.049, *ϕ* = 0.22. In addition, fewer patients with RC were diagnosed with PTSD compared to patients with NRC, *χ*(2) = 6.46, p=.040, *ϕ* = 0.23. With regard to the EDE‐Q subscales, patients with RC reported higher *weight concern* compared to patients with NRC on admission, *t*(112) *=* 2.89, p=.032, *d =* 0.54. No further differences were found (see Table 3).

As described above, a binary logistic model with the backward likelihood method was conducted. The final model was statistically significant, *χ* ^2^(3) = 22.58, p<0.001., resulting in an acceptable amount of explained variances (Nagelkerke's *R* ^2^ = 0.240). The overall percentage of accuracy in classification was 68.4%. Of the four independent variables, three were included in the final model, of which each significantly predicted RC: the EDE‐Q subscale *weight concern* (p=.001), anxiety disorder (p=.025) and PTSD (p=.003). Higher *weight concern* on admission enhanced the likelihood for RC, OR = 1.68 (95%–CI [1.23, 2.29]), while being diagnosed with anxiety disorder reduced the likelihood for RC, OR = 0.39 (95%–CI [0.17, 0.88]), as did being diagnosed with PTSD, OR = 0.21 (95%–CI [0.08, 0.60]). With an increase of 1.33 points in the *weight concern* subscale on admission, the odd for RC doubled. Patients who were diagnosed with anxiety disorders had a 2.56 higher probability for NRC, while those diagnosed with PTSD had a 4.76 higher probability.

## 4 Discussion

The aim of the present study was to provide updated data about the effectiveness of ED treatment in departments of psychosomatic medicine and psychotherapy in Germany.

Within the AN patient group, the eating pathology significantly decreased from admission to discharge. This decrease remained stable until the 1‐year follow‐up assessment. This finding is consistent with (Billman Miller et al. 2024) who reported EDE‐Q reductions from intake to discharge in a similar magnitude for both AN and atypical AN in young adults.

*N* = 51 (33.8%) of the patients with AN reliably changed on the EDE‐Q mean score between admission and the 1‐year follow‐up assessment. This result is in line with the above mentioned study of (Schlegl et al. 2014) who reported a clinical significant change of eating pathology in 35.6% of patients with AN at discharge.

In patients with AN BMI increased significantly from admission (*M* = 15.4 kg/m^2^) to discharge (*M* = 16.6 kg/m^2^) and even further until the 1‐year follow‐up assessment (*M* = 17.5 kg/m^2^). This is comparable to (Tagay et al. 2011) who reported an increase of BMI from 15.15 kg/m^2^ on admission to 17.49 kg/m^2^ on discharge. At the 1‐year follow‐up assessment, *N* = 22 (24.2%) of the patients with AN had a BMI > 18.5 kg/m^2^ and therefore within the normal range. That is also comparable to (Schlegl et al. 2014) who reported 24.8%. Kächele et al. (2001) reported a remission rate of 33% in patients with AN 2.5 years after admission. Remission rates tend to increase over time (e.g., 40% after 20 years) (Fichter et al. 2017). However, the definition of remission varies across studies, thereby compromising comparability and underscoring the need for consistent definitions (Khalsa et al. 2017).

In contrast to (Schlegl et al. 2014), depression did not predict RC in patients with AN. In our study, RC was negatively predicted by somatoform disorders and positively predicted by shape concerns on admission. Prior studies indicated that a majority of young people with AN have primarily shape (60%), instead of weight concerns (30%) (Byrne et al. 2015). Therefore, the treatment of body image distortions for example by means of cognitive restructuring, exposition therapy or body focused self‐care, may be particularly important in AN treatment (McLean and Paxton 2019).

Similar to patients with AN, the eating pathology of patients with BN decreased between admission and discharge and remained stable until the 1‐year follow‐up assessment. A total of *N* = 52 (43.3%) patients with BN achieved a RC in eating pathology between admission and the 1‐year follow‐up assessment. This is comparable to (Diedrich et al. 2018) who reported clinical significant changes in 43.2% of patients with BN.

The frequency of compensatory behaviour in patients with BN decreased about 50% from admission to the 1‐year follow‐up assessment. The number of people reporting *severe or extreme* BN severity decreased from 26% to 11% at the 1‐year follow‐up assessment. The frequency of binge eating decreased from admission to the 1‐year follow‐up assessment. At the 1‐year follow‐up assessment, *n* = 57 (54%) of patients with BN reported to have less than 1 binge eating/week, therefore not fulfilling the diagnostic criteria for BN disorder (F50.2). A review of 28 ED treatment outcome reviews indicated mean posttreatment abstinence rates of binge eating between 50% and 70% (Richards et al. 2000). Kächele et al. (2001) reported remission rates of only 25% 2.5 years after treatment, but remission was operationalised as a combination of fewer than 2 binge eating/week, the abstinence of compensatory behaviour and significant reductions in weight and shape concern. As mentioned above, there is a need for consistent definitions of remission rates in eating disorders. Increased remission rates have been reported for 6 years (50%) and 21 years (70%) follow‐up assessments (Castellini et al. 2011; Quadflieg and Fichter 2019).

In our study, significant predictors for RC in BN were anxiety disorders, PTSD and weight concern. According to the cognitive model of BN as proposed by (Fairburn et al. 2003), weight concerns are a core symptom of patients with BN and drivers for compensatory behaviours. In contrast to other aspects of eating pathology, weight concerns mainly develop due to environmental factors, such as advertisements that postulate thinness as desirable, or bullying experiences (T. Wade et al. 1998; Day et al. 2022). Patients with BN with comorbid anxiety disorders had a doubled probability for NRC, and those with comorbid PTSD nearly five times higher probability. The prevalence of anxiety disorders in BN has been previously reported between 16% for current and 64% for lifetime comorbidity (Jaite et al. 2013; Bulik et al. 1996). Early‐onset anxiety disorders are thought to be a potential pathway to the development of BN, yet there is a need for more research on that topic (Swinbourne and Touyz 2007). PTSD is a prominent comorbidity of patients with BN (37%), and many patients experienced victimization before the onset of BN (Dansky et al. 1997). The coexistence of PTSD symptomatology is associated with severe ED pathology and premature treatment termination (Trottier 2020). Therefore, it is of importance to thoroughly explore anxiety disorder and PTSD symptoms in patients with BN.

This manuscript provides updated data on the effectiveness of ED treatment in departments of psychosomatic medicine and psychotherapy in Germany. The results are comparable to prior multi‐and mono‐centred studies in Germany (Tagay et al. 2011; Schlegl et al. 2014; Diedrich et al. 2018; Kächele 1999; Kächele et al. 2001). Yet, health care service conditions for patients with EDs vary greatly between European countries (Richard 2005a; Monteleone et al. 2023). For example, most of the patients with BN in Europe are treated in outpatient settings, with the exception for German‐speaking countries (Richard 2005a). Yet, our results provide evidence that i.e. patients with BN could benefit from inpatient and day‐hospital treatment with a RC over 43% and partial remission of 54% 1 year after discharge. Results from a large Swedish population study revealed an association between treatment duration of patients with AN and a decreased probability for relapse (Andersson et al. 2023). In our study, treatment duration was no significant predictor of RC in patients with AN. With an average of 73 days the treatment duration of patients with RC was on a descriptive level higher than those of the patients without RC who stayed on average for 62 days. Moreover, the duration was comparable to global data on length of stay for patients with AN (Kan et al. 2021). As observed in adolescents with AN a quick transfer from inpatient to day‐hospital treatment might equally improve eating pathology than resting in inpatient treatment (Herpertz‐Dahlmann et al. 2014). Moreover, in accordance with the abovementioned “step‐down” approach, outpatient aftercare might have a beneficial impact on eating pathology (Giel et al. 2021). Unfortunately, we could not provide data about the treatment of the patients following discharge, which aggravates the interpretation of potential associations between treatment dose and outcome.

Further limitations are that although the advanced age and high unemployment rates suggest that the patient group was particularly burdened, the study design lacks information about the degree of chronicity of the patients and former treatments. In addition, since the majority of patients underwent inpatient treatment, a statistical comparison to day‐hospital treatment was not possible. Data on individual treatment history could provide important insights and should be considered in future research.

Moreover, the BMI at the follow‐up assessment was self‐reported that might have been less reliable at T2 than at the first two assessments. There was a high drop‐out rate in both ED subsamples. Future studies should provide more incentives for participation at the follow‐up assessment. We chose to conduct the LOCF method to deal with missing values (including those that were lost to follow‐up) in order to not overestimate the changes from admission to discharge, yet that also limits the interpretability of the follow‐up assessment.

There are also advantages and implications that could be derived from this manuscript. Due to the combination of statistical analyses on group and on individual levels, conclusions about vulnerable patient groups and implications for future treatment strategies can be derived. Of particular importance is the management of comorbid mental disorders, namely somatoform disorders in patients with AN and anxiety disorders and PTSD in patients with BN. As mentioned above, inpatient/day hospital treatment of EDs in Germany is recommended if the patients experience complex comorbidity with other mental disorders. Individual treatment might subsequently include additional disorder‐specific treatment elements for comorbid disorders. Therefore, future studies on the effectiveness of inpatient/day hospital ED treatment should also consider and analyse the impact on comorbid symptomatology. In addition, the treatment of body image distortions, shape and weight concerns seem to be especially beneficial. Although ED therapy varies largely in Europe, our results are comparable not only to national, but also to former European studies on the effectiveness of ED treatment (Richard 2005b) and therefore contribute to an update of effectiveness data. Future international studies are warranted that provide longitudinal data on the patients' way into specialised ED therapy, the effectiveness of inpatient and day‐hospital treatment and the years after discharge.

## 5 Conclusion

This multi‐center study on the effectiveness of inpatient and day‐hospital treatment of patients with AN and BN in German university departments of psychosomatic medicine and psychotherapy demonstrates RC in ED pathology in 33.8% of patients with AN and 43.3% of patients with BN, as well as partial remission in 24% patients with AN and 54% patients with BN, 1 year after discharge. These results are comparable to former multi‐center and single‐center studies on inpatient treatment. There is a need for international multi‐center studies assessing longitudinal data about the effectiveness and sustainability of ED treatment.

## Funding

The authors have nothing to report.

## Ethics Statement

The study was initially approved by the Ethics Committee of the medical faculty of the Ruhr‐University Bochum on October 17, 2018 (ID: 18‐6388, this approval was subsequently confirmed by the Ethics Committees of the participating universities). All patients gave written informed consent for their participation in the study.

## Conflicts of Interest

The authors declare no conflicts of interest.

## Data Availability

The data that support the findings of this study are available on request from the corresponding author. The data are not publicly available due to privacy or ethical restrictions. The datasets presented in this article are not readily available because the European General Data Protection Regulation (GDPR) does not allow to share personal data of patients publicly (https://gdpr.eu). The ethics commissions of all of the study centers have approved the study under the condition that even the transfer of data from the German sites to the Austrian PI (SD) can only take place according to specific security regulations. Requests to access the datasets should be directed to stephan.doering@meduniwien.ac.
